# Rhinovirus Infections among Hematopoietic Stem Cell Transplant Recipients: A Pre-Transplant Dilemma?

**DOI:** 10.3390/v14020267

**Published:** 2022-01-28

**Authors:** Sébastien Barral, Aline Mamin, Carole Dantin, Stavroula Masouridi-Levrat, Yves Chalandon, Laurent Kaiser, Diem-Lan Vu

**Affiliations:** 1 Faculty of Medicine, University of Geneva, 1206 Geneva, Switzerland; Sebastien.Barral@etu.unige.ch (S.B.); yves.chalandon@hcuge.ch (Y.C.); laurent.kaiser@hcuge.ch (L.K.); 2 Laboratory of Virology, Division of Laboratory Medicine, Division of Infectious Diseases, Geneva University Hospitals, 1205 Geneva, Switzerland; aline.mamin@hcuge.ch; 3 Division of Hematology, Department of Oncology, Faculty of Medicine, Geneva University Hospitals, 1205 Geneva, Switzerland; carole.dantin@hcuge.ch (C.D.); stavroula.masouridi@hcuge.ch (S.M.-L.); 4 Department of Infectious Diseases, Geneva University Hospitals, 1205 Geneva, Switzerland; 5 Center for Emerging Viruses, Geneva University Hospitals, 1205 Geneva, Switzerland

**Keywords:** respiratory virus, rhinovirus, stem cell transplantation, shedding

## Abstract

Respiratory viral infections (RVIs) in allogeneic hematopoietic stem cell transplant (allo-HSCT) recipients can be of concern due to the patients’ depressed immune status, but few data are available about the significance of a pre-transplant positive testing. In this retrospective observational study, we analyzed a cohort of patients that were transplanted between 1 January 2010 and 31 October 2019 in the Geneva University Hospitals with at least one RVI before or after transplantation. At least one RVI was detected in 319/533 (63.5%) transplanted patients. Rhinoviruses were most frequently identified (37%), followed by human coronaviruses (17.1%), parainfluenza viruses (13.9%), and influenza viruses (9.9%). First infection in the post-transplant period occurred at a mean time of 334 days (SD 338). Specific analysis of a subgroup of 65 patients with pre-transplant RVIs was performed. Among them, 39 (59%) patients had symptoms and 14 (21.2%) had a lower respiratory tract infection. Four patients (6.1%) (three rhinovirus and one influenza) needed an intensive care unit admission, of which, three (4.5%) (two rhinovirus and one influenza) were intubated. The patient with influenza infection diagnosed the day of the transplantation died within the first 30 days of the infection. Two patients with rhinovirus infection died within 3 months of unrelated causes. Our data show that rhinovirus infections are predominant in allo-HSCT patients, including among pre-transplant infections; however, mortality due to pre-transplant RVI is low and was only clearly identified in one patient with influenza infection. RVI within the month preceding allo-HSCT is not associated with direct morbidity or mortality in this cohort.

## 1. Introduction

Respiratory viral infections (RVIs) are frequent and affect all populations. The SARS-CoV-2 pandemic is a perfect example of the ability of these viruses to be efficiently transmitted from one individual to another, within the community, and through social interactions. Human rhinoviruses (HRVs) are recognized as the most common cause of acute respiratory infections. They are usually characterized by a syndrome familiarly called a common cold [1], which spontaneously resolves within a few days. A large epidemiological study confirmed that HRVs rarely cause severe disease [2]. Yet, recent studies tend to identify more severe cases of HRV infections in some specific populations. In a prospective study of 590 children hospitalized for acute respiratory infection, HRV infections represented 33.4% of cases and more than half of patients (55.8%) had lower respiratory tract infection (LRTI) [3]. Lu et al. observed a 10% rate of viremia among hospitalized patients with HRV infection [4]. Patients with hematologic disease also seem to be more susceptible to severe HRV infections. A prospective study on 110 patients with HRV infection and hematologic malignancy showed that 29% had LRTI, among which 25% had bacterial co-infection [5]. Another prospective cohort study comparing HRV and FLU pneumonia among adult patients in intensive care units (ICUs) observed a high but similar 28 day mortality (29.6% vs. 35.3%, respectively, *p* = 0.61) between groups and HRV infections occurred more often in immunosuppressed patients (81.5% vs. 33.3%, *p* < 0.001) [6].

Many other respiratory viruses can cause syndromes similar to the common cold, including human coronavirus (HCoV), respiratory syncytial virus (RSV), parainfluenza (PIV), influenza viruses (FLU), human metapneumovirus (hMPV), adenovirus (ADV) and bocaviruses [1], and a definitive microbiological diagnostic is essential to investigate the role of each virus in high-risk settings.

Allogeneic hematopoietic stem cell transplant (allo-HSCT) recipients receive several courses of chemotherapy and a conditioning regimen leading to a transient complete aplasia. Patients also receive immunosuppressive drugs for months or even years after transplantation, to prevent or treat graft-versus-host disease (GvHD). As a result, transplanted patients are at increased risk of infections with potential severe complications. Among this population, RVI can be more severe than a common cold, leading to respiratory failure or even death [7]. Any infection before or immediately after the transplantation among these patients is not anecdotal and must, therefore, be particularly investigated, as it can be a major cause of transplant-related mortality [8].

A retrospective matched case-control study of HRV infections among 141 allo-HSCT adult patients pointed out that HRV infections are common after transplantation and are associated with an increase in rehospitalizations of any cause (46.8% versus 24.5%), but not with other outcomes such as ICU admission or mortality [9]. In contrast, another retrospective study among adult allo-HSCT patients with HRV infection found a 90 day overall mortality rate of 6% and 41% in cases of upper and lower respiratory tract infection, respectively (*p* < 0.001). HRV pneumonia had a mortality rate comparable to that of pneumonia with other respiratory viruses (RSV, PIV, and FLU) [10].

Pre-transplant respiratory viral infections are of particular concern, as they can challenge the transplantation procedure. Yet, in a retrospective study among 585 pediatric patients benefiting from allo-HSCT, transplant delay for patients with a positive screening test for cytomegalovirus, ADV, RSV, FLU, PIV, or hMPV led to a better overall survival rate (79% versus 54%) and a reduced transplant-related mortality rate (7% versus 26%) [11]. Another retrospective multicentric study including 402 adult and pediatric patients with pre-transplant HCoV infections found similar results; HCoV lower respiratory tract infections led to a higher three-month overall mortality (16%) compared to HCoV upper respiratory tract infections (7%) [12]. In contrast, in their matched case-control study, Abandeh et al. found that patients with pre-transplant HRV infection had non-significantly worse outcomes than those without infection [9]. 

In our study, we described the epidemiology of RVI in a cohort of adult allo-HSCT patients in the Geneva University Hospitals (HUG) during the pre-COVID-19 pandemic era. We focused on pre-transplant RVI with HRV to determine the clinical significance of a pre-transplant HRV infection. 

## 2. Material and Methods

### 2.1. Setting, Study Population, and Design

In this retrospective observational descriptive study, we analyzed allo-HSCT recipients transplanted between 2010 and 2019 at the HUG with at least one RVI. We collected all samples detected positive by real-time (reverse-transcription) polymerase chain reaction (RT-PCR) assay from the database of the HUG Laboratory of Virology from 1 January 2010 to 31 October 2019. The included respiratory viruses were: HRV, enterovirus, parechovirus, HCoV (OC43, HKU1, NL62 and 229E types), RSV (A and B), PIV (1, 2, 3 and 4), FLU (A and B), hMPV, ADV, and bocavirus. The data were crossmatched with the institutional database of the HUG Service of Hematology to identify adult allo-HSCT recipients over 16 years old with at least one RVI occurring from one month before transplantation up to the patients’ last follow-up within a prospective cohort of allo-HSCT recipients (CCER n°14–220). The patients studied were allo-HSCT recipients with at least one RVI. Screening for respiratory viruses was symptom-driven except in the pre-transplant setting, where a systematic pre-transplant screening was implemented about 1 month before transplantation since late 2014. Yet, according to our internal procedures, patients have a pre-transplant evaluation about one month before HSCT to rule out any contra-indication for transplantation. This evaluation notably includes a pre-transplant infectious disease evaluation, including a systematic screening for respiratory viruses, to ensure there are no ongoing infections that could be harmful to the transplant success. Most patients are already immunocompromised by the underlying disease and sequential courses of chemotherapy at this time and are thus at an increased risk for opportunistic infections.

Medical information was collected from the HUG institutional database and from the database of the HUG Service of Hematology. Demographical data, clinical manifestations, and complications were included. 

### 2.2. Microbiological Methods

During the study period, several RT-PCR assays were conducted. From 1 January 2010 to 26 June 2013, in-house RT-PCR assays were applied for ADV, FLU A/B, PIV 1 to 3, hMPV, RSV, and a generic picornavirus recognizing HRV and enterovirus. From 23 August 2011 to 26 June 2013, HCoV HKU1, NL63, and OC43 and 229E were added to the panel. These in-house RT-PCR assays were designed according to in silico alignment and validated using clinical studies and quality controls. From 26 June 2013 to 23 August 2019, a commercial kit FTD-2–64 Respiratory Pathogen 21 (Fast Track Diagnostics, Esch-sur–Alzette, Luxembourg) was used for detecting the following viruses: ADV, FLU A/B, PIV 1 to 4, hMPV, RSV, HCoV HKU1, NL63, OC43 and 229E, parechovirus, a specific enterovirus, and the generic rhinovirus. Finally, on 23 August 2019, some targets of the FTD panel (FLU A/B, hMPV, RSV, PIV 1 and 3 and the generic rhinovirus) were replaced by an in-house (RT-) PCR assay. The mixes were commercially produced by Kaneka Eurogentec SA (Seraing, Belgium) according to the same, or updated, designs used before the FTD panel. In summary, ADV, FLU A/B, hMPV, PIV 1–3, RSV and HRV, and enterovirus were screened during the entire study period; HCoV HKU1, NL63, and OC43 and 229E were screened from 23 August 2011 to the end of the study period; and Bocavirus and PIV 4 were screened from 26 June 2013 to the end of the study period.

### 2.3. Definitions

An RVI was defined as a sample determined positive by RT-PCR assay for one of the studied viruses. Collected samples were naso-pharyngeal swabs/aspirations (NPS/NPA) or bronchoalveolar lavages/liquids (BAL), reflecting upper and lower respiratory sites, respectively. If more than one respiratory virus was detected in the same sample, it was considered as a viral co-infection. Persistence of a positive test for a particular virus was considered as the same RVI episode if there was no negative test between two positive tests or if both positive tests were not separated by more than one month. A longer time period was permitted according to the clinical situation, particularly in the case of a high immunosuppression for GvHD. We used the time period elapsed between two positive tests for the same pathogen to determine the shedding duration. If the conditions mentioned above were not respected, the RVIs were considered as distinct episodes. 

We also characterized the site of the respiratory tract infection. An upper respiratory tract infection (URTI) was defined as a positive RT-PCR result in an NPS/NPA with or without associated symptoms and with no arguments for LRTI. A lower respiratory tract infection was defined as an RVI with at least one of the following criteria: a positive RT-PCR result in a respiratory sample (NPS/NPA and/or BAL) with a compelling pulmonary infiltrate on a CT scan without alternate diagnosis, or a positive RT-PCR result attributed to an RVI by the clinicians or radiologists, with or without typical symptoms for an LRTI. BALs were performed at the physician’s discretion according to the clinical situation.

### 2.4. Statistical Analyses

Comparisons between continuous variables were performed using Mann–Whitney and Kruskal–Wallis tests, and *p* < 0.05 was considered statistically significant. Statistics were performed by Stata/IC 13.1 (StataCorp, College Station, TX, USA).

## 3. Results

### 3.1. General Overview

Between 1 January 2010 and 31 October 2019, 533 adult patients benefited from an allo-HSCT at the HUG. Of those 533 patients, 20 had multiple transplantations, including 18 with two transplantations and 2 with three transplantations. At least one RVI was detected before or after transplantation in 319 (63.5%) patients. The first post-transplant infection occurred at a mean time of 334 days (SD ± 338) and a median number of episodes per patient of two (range 1–18) was observed. The population of patients was characterized by a mean age of 48.8 years (SD ± 13.66) at the time of the transplantation with the majority being male patients (*n* = 204; 64%).

**Figure 1:**
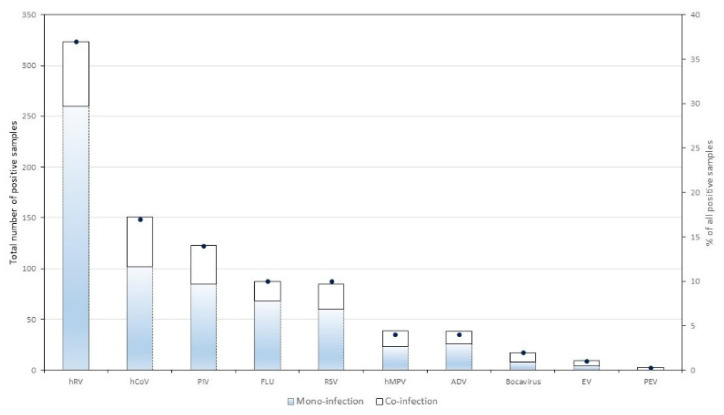
Positive samples of respiratory viruses. Bar charts represent the absolute number of positive samples for each screened respiratory virus and are divided according to mono- and co-infections. Dots represent the proportion of positive samples among all positive samples. HRV—human rhinovirus; HCoV—human coronavirus; PIV—parainfluenza virus; FLU—influenza; RSV—respiratory syncytial virus; hMPV—human metapneumovirus; ADV—adenovirus; EV—enterovirus; PEV—parechovirus.

In total, 874 distinct RVI episodes were identified among 319 patients, including 231 (26%) co-infections with more than one virus detected in the same sample. The RVIs were attributed to 323 HRV infections (36.9%), 151 HCoV (17.2%), 123 PIV (14%), 87 FLU (9.9%), 85 RSV (9.7%), 39 hMPV (4.5%), 38 ADV (4.3%), 17 bocavirus (1.9%), 9 enterovirus (1%) and 3 parechovirus (0.3%). Most RVIs were exclusively identified in an upper respiratory sample (*n* = 809; 92.5%), while 34 (4%) were identified both in upper and lower respiratory samples, and 32 (3.7%) were identified in a lower respiratory sample only (Figure 1; Supplementary Table S1). We calculated a mean shedding duration of 26.6 days (SD ± 19) among 134 episodes in 112 patients for whom the information was available. By analyzing only post-transplant RVIs and comparing infections occurring within 6 months and those occurring more than 6 months after transplantation, we found a significant difference in the shedding duration, with a median of 24 days (IQR 16–43) for RVIs occurring within 6 months versus 17 days (IQR 13–28) for RVIs occurring beyond 6 months (*p* = 0.017, univariate analysis). 

### 3.2. Pre-transplant HRV Infections

We analyzed a subgroup of patients with RVI occurring within the month before their transplantation. The group consisted of 65 patients with a mean age of 48 years (SD ± 14.4) and the patients were predominantly of male sex (*n* = 38; 58.5%) (Table 1).

Table 2 shows the details of the 66 (7.4%) distinct RVIs found. The most frequently detected viruses were HRVs (*n* = 30; 45.4%), followed by HCoVs (*n* = 14; 20.9%), FLUs (*n* = 10; 14.9%), PIVs (*n* = 3; 4.5%), RSVs (*n* = 3; 4.5%), hMPVs (*n* = 3; 4.5%), ADVs (*n* = 2; 3%), enteroviruses (*n* = 1; 1.5%), and bocaviruses (*n* = 1; 1.5%). Only four episodes of co-infections were observed: two HCoV infections (with bocavirus and HRV, respectively), one RSV (with HCoV and PIV), and one HRV (with PIV). Pre-transplant RVI occurred at a median time of 14.5 days (IQR 8.3–22.8) and, among 56 patients, it persisted for a median duration of 21.5 days (IQR 14.5–39.3). Specifically, HRV infection occurred at a median time of 10.5 days (IQR 8–22.8) before transplantation. Among 30 patients with available data, the infection persisted for a median duration of 30.5 days (IQR 20.3–47.5). Of note, in 22 among 66 pre-transplant RVI episodes, the RT-PCR test was negative shortly before transplantation.

According to clinical information, 39 (58%) patients had symptoms and 14 (21.2%) had LRTIs. Among HRV infections, 18 (60%) patients were symptomatic and only 4 (13.3%) developed a clinical and radiological pneumonia, all with favorable outcome. A total of 21 (31%) patients with any pre-transplant RVI benefited from medical treatment: 12 (17.9%) received antivirals, including 9 (90%) influenza episodes treated by neuraminidase inhibitors (oseltamivir), 2 (66.7%) RSV episodes, 1 HRV (3.2%), and 1 hMPV (33.3%) treated by nucleoside inhibitors (ribavarin). Twelve (17.9%) received immunoglobulins: six (19.4%) for HRV, three (100%) for RSV, two (14.3%) for HCoV and one (33.3%) for hMPV. Finally, three (4.5%) benefited from both. 

According to clinical outcomes, four patients (three HRV and one FLU A, 6%) required an ICU admission, of which three (two HRV and one FLU A, 4.5%) were intubated. Among three HRV-infected patients with ICU admission, one was admitted and intubated to perform a diagnostic bronchoalveolar lavage, the second had diffuse bleeding, including cerebral bleeding, and necessitated intubation for impaired alertness, and the third was admitted for acute kidney injury necessitating dialysis. In rare cases, the patient died shortly after the transplantation; one patient with FLU infection diagnosed the day of the transplantation died within the first 30 days of the infection and two patients with HRV and one patient with enterovirus infection died within 3 months. Nevertheless, none of these deaths were attributable to the HRV/enterovirus infection but were attributed to severe pulmonary fungal infection/severe bleeding, diffuse bleeding, and severe digestive GvHD, respectively. Only the first patient had a virus (HRV) detected in BAL.

## 4. Discussion

In our study, at least one RVI occurred in 60% of allo-HSCT recipients. Rhinoviruses were predominant, accounting for almost 40% of all RVIs, and we found few positive lower respiratory tract samples (<10% of all positive). RVI is thus a prevalent condition in allo-HSCT recipients but seems to remain benign in the majority of cases; however, we did not specifically analyze the total number of BALs performed and therefore could not provide a true rate of positivity. In addition, more than one virus was detected in almost 30% of RVIs, probably reflecting the impaired cellular immunity.

The first episode of post-transplant RVI occurred at a mean time of 334 days (SD ± 338), which is quite distant from the HSCT and could be explained by the strict hospitalized protective conditions during the first weeks after transplantation and the precautions taken during the first months after hospital discharge. We identified a slightly longer shedding duration when RVI occurred within 6 months after the transplantation compared to beyond (29.8 versus 22.8 days). This is also probably due to the patients’ immunosuppressive treatment and the lack of a sufficient immune reconstitution to efficiently clear the infection within the first months [13]. Interestingly, Pinana et al. observed a higher risk of mortality among patients with RVI occurring within 6 months after transplantation [14]. Of note, no difference in shedding duration was observed between RVIs occurring within compared to after 3 months after transplantation.

There are currently no clear directives existing for the management of pre-transplant RVI. A positive RT-PCR test for an RVI is not systematically followed by a delay of transplantation. The response depends on the clinician’s decision, weighing the patient’s disease severity and the risk to undergo the transplantation with the risk to wait for viral clearance. We identified 66 pre-transplant RVIs among 65 patients, which represent less than 10% of all RVIs identified in the study. In addition, 30% of pre-transplant RVI patients were already cleared shortly before allo-HSCT, which is probably underestimated, as a control was not systematically performed. Severe outcomes defined as ICU admission, intubation, and/or death were observed only in four, three, and four patients, respectively. Among them, all but one had HRV/enterovirus infection. The over-representation of HRV in severe cases is questionable, but after an in-depth analysis of patients’ clinical records, severe outcomes were found to be independent of HRV infection, potentially challenging the high morbidity and mortality rates associated with HRV infections in former studies [10]. In contrast, one patient with a FLU infection that was discovered the day of the transplantation died of the viral infection within 30 days. Interestingly, there was a 20% rate of LRTI in the pre-transplant subgroup, which is probably biased by the CT scan performed before transplantation as a routine procedure. As it was revealed for COVID disease, there could be a discordance between radiographic findings and RVI clinical manifestations; in a retrospective study among 139 hospitalized adult patients with SARS-CoV-2, 10 asymptomatic patients had lung alterations on chest CT scans [15].

The rate of LRTI among pre-transplant HRV infection is the lowest (13%) compared to other respiratory viruses (66% for RSV and PIV, 40% for FLU, and 33% for hMPV). The results observed are similar to those reported in previous studies [7,16,17]. Specifically, Waghmare et al. observed a similar proportion of HRV progression to LRTI and identified low lymphocyte count, low albumin, positive cytomegalovirus serostatus, recipient statin use, and steroid use (≥2 mg/kg/day) as risk factors for such progression [7]. Another study observed a higher 3 month mortality rate in cases of HRV LRTI compared to HRV URTI (41% vs. 6%) [10]. In our cohort, among four HRV infected patients with LRTI, only one died within 3 months from severe fungal infection and severe bleeding. Although we cannot confirm that HRV infection did not indirectly influence the poor outcomes of patients, e.g., by favoring fungal co-infection, our analysis highlights that allo-HSCT recipients are at an increased risk of multiple infectious and non-infectious complications associated with several levels of severity, complicating the causal link with severe outcomes. In term of overall mortality, 46.9% of patients with a pre-transplant RVI died, which is concordant with the mortality rate observed in the whole cohort of allo-HSCT recipients [18]. 

The retrospective nature of the study is its main limitation, leading to a potential lack of clinical and biological data. Nevertheless, the main outcomes of ICU admission, intubation, and death should be accurate as systematic pre-transplant screening for RVI has been implemented since late 2014 in our institution. There were two changes in the use of RT-PCR assays during the study period, potentially leading to a variability in the positivity rate, but according to the data and congruent with the techniques validation, we are confident that there was no significant variability that could have influenced the study. The shedding durations could also be biased by the absence of systematic control RT-PCR testing and because RT-PCR tests were performed at the physicians’ discretion. Due to the low number of outcomes among patients with pre-transplant RVI, we could not perform multivariable statistical analysis to determine factors that can influence poor outcome (e.g., underlying disease, GvHD, type and dose of immunosuppressive drugs). Future prospective studies can attempt to find a correlation with lymphocyte counts. Nevertheless, the study relied on a cohort of allo-HSCT patients and molecular virological data, which both provide reliable results to assess the significance of RVI among allo-HSCT recipients.

## 5. Conclusions

In conclusion, this study highlights the predominant role of HRV infection among RVI in allo-HSCT recipients, including among pre-transplant infections. We observed that severe attributable outcomes are rare in cases of pre-transplant HRV infection, as is the case with other respiratory viruses, supporting the strategy of not systematically delaying transplantation. Further investigations should be performed to assess a potential indirect role of pre-transplant RVI in severe outcomes.

## Figures and Tables

**Table 1:** Characteristics of patients with pre-transplant RVI.

	Total (*n* = 65)
**Demographics**	
Sex (male), *n* (%)	38 (58.5)
Age, mean (SD)	48 (14.4)
**Allo-HCT considered in the analysis, *n* (%)**	
First	56 (84.8)
Second *	10 (15.2)
**Transplant source, *n* (%)**	
Bone marrow	11 (16.9)
Peripheral blood cells	54 (83.1)
**Underlying disease, *n* (%)**	
Acute myeloid leukemia	34 (52.3)
MDS/MPS	10 (15.4)
Lymphoid malignancy	9 (13.8)
Acute lymphoid leukemia	6 (9.2)
Multiple myeloma	4 (6.1)
Chronic leukemia	2 (3.1)
**Disease risk, *n* (%)**	
Low	2 (3.1)
Intermediate	40 (61.5)
High	21 (32.3)
Very high	2 (3.1)
**EBMT Risk score, mean (SD)**	3.75 (1.33)
**Donor match, *n* (%)**	
Matched-related	20 (30.8)
Matched-unrelated	26 (40)
Mismatched unrelated	8 (12.3)
Haplo-identical	11 (16.9)
**Conditioning, *n* (%)**	
Myeloablative conditioning	20 (30.3)

* One patient had a pre-transplant RVI before first and second allo-HSCT. EBMT risk score from 1–7. RVI—respiratory viral infection; MDS/MPS—myelodysplasic syndrome/myeloproliferative syndrome; SD—standard deviation.

**Table 2:** Characteristics of RVI occurring within the month preceding transplantation.

Pre-Transplant (<30 Days)	Total	HRV	HCoV	FLU	hMPV	PIV	RSV	ADV	EV
Total, n (%)	66	30 (45.5)	14 (20.9)	10 (14.9)	3 (4.5)	3 (4.5)	3 (4.5)	2 (3)	1 (1.5)
Delay from Tx, Median day (IQR)	15 (9–23)	10.5 (8–22.8)	21 (13.3–22.8)	17.5 (9.3–21.5)	21 (17–25)	15 (13–15.5)	13 (13–18)	19.5 (17.8–21.3)	24
Shedding, Median day (IQR)	21.5 (14.5–39.3)	30.5 (20.3–47.5)	24 (18–30.8)	11 (4–14.3)	17,5 (15.3–19.8)	21 (14–29.5)	57 (32–64.5)	8	20
Symptoms, n (%)	39 (59)	18 (60)	6 (43)	8 (80)	1 (33.3)	2 (66.7)	3 (100)	0	1 (100)
Lower respiratory tract involvement, n (%)	14 (21.2)	4 (13.3)	0	4 (40)	1 (33.3)	2 (66.7)	2 (66.7)	0	1 (100)
Intensive care admission, n (%)	4 (6.1)	3 (13.3)	0	1 (10)	0	0	0	0	0
Intubation, n (%)	3 (4.5)	2 (6.7)	0	1(10)	0	0	0	0	0
Treatment all, n (%)	21 (31.8)	7 (23.3)	2 (14)	8 (80)	1 (33.3)	0	3 (100)	0	0
Antivirals, n (%)	12 (18)	1 (3.3)	0	8 (80)	1 (33.3)	0	2 (66.7)	0	0
Immunoglobulins, n (%)	12 (18)	6 (20)	2 (14)	0	1 (33.3)	0	3 (100)	0	0
Overall mortality, n (%)	31 (46.9)	12 (40)	8 (57)	5 (50)	2 (66.7)	1 (33.3)	2 (66.7)	0	1 (100)
30 day overall mortality, n (%)	1 (1.5)	0	0	1 (10)	0	0	0	0	0
3 month overall mortality, n (%)	4 (6.1)	2 (6.7)	0	1 * (10)	0	0	0	0	1 (100)

* One patient died from FLU infection within 30 days after transplantation and was thus also counted in the 3 month overall mortality. RVI—respiratory viral infection; HRV—human rhinovirus; HCoV—human coronavirus; PIV—parainfluenza virus; FLU—influenza; RSV—respiratory syncytial virus; hMPV—human metapneumovirus; ADV—adenovirus; Boca—bocavirus; EV—enterovirus; PEV—parechovirus; *n*—number; SD—standard deviation; Tx—transplantation; IQR—interquartile range.

## Data Availability

The data presented in this study are available on request from the corresponding author. The data are not publicly available due to the absence of specific consent obtained through the general inform consent form that the patients have signed.

## Supplementary Materials

The following supporting information can be downloaded at: https://www.mdpi.com/article/10.3390/v14020267/s1. Table S1: RVI in HSCT patients: global results.
