# Supplementary material for: Rhinovirus Infections among Hematopoietic Stem Cell Transplant Recipients: A Pre-Transplant Dilemma?
Source: Viruses. 2022 Jan 28;14(2):267. doi: 10.3390/v14020267 (PMC8879386; doi:10.3390/v14020267)
Supplement: Supplementary file 1 [file viruses-14-00267-s001.zip › viruses-1537874-supplementary.pdf]

**Table S1: RVI in HSCT patients: global results.**

| | Total | hRV | hCOV | PIV | FLU | RSV | hMPV | ADV | Boca | EV | PEV |
|---|---|---|---|---|---|---|---|---|---|---|---|
| **Total, n (%)** | **875** | 323 (37) | 151 (17) | 123 (14) | 87 (10) | 85 (10) | 39 (4) | 38 (4) | 17 (2) | 9 (1) | 3 (0.3) |
| **2009-10** | **3** | 2 (66.7) | 0 | 0 | 0 | 0 | 1 (33.3) | 0 | 0 | 0 | 0 |
| **2010-11** | **23** | 10 (43.3) | 0 | 5 (22) | 1 (4.3) | 4 (17.3) | 0 | 3 (13) | 0 | 0 | 0 |
| **2011-12** | **58** | 28 (48) | 12 (21) | 3 (5.2) | 5 (8.6) | 6 (10.3) | 2 (3.4) | 2 (3.4) | 0 | 0 | 0 |
| **2012-13** | **77** | 31 (40.3) | 8 (10.4) | 12 (15.6) | 14 (18.1) | 8 (10.4) | 3 (3.9) | 1 (1.3) | 0 | 0 | 0 |
| **2013-14** | **142** | 43 (34.5) | 24 (16.6) | 19 (13.1) | 13 (9) | 17 (11.7) | 9 (6.2) | 8 (5.5) | 5 (3.4) | 3 (33) | 1 (33) |
| **2014-15** | **133** | 32 (25.6) | 35 (26.3) | 20 (15) | 13 (9.8) | 17 (12.8) | 6 (4.5) | 6 (4.5) | 3 (2.3) | 1 (1) | 0 |
| **2015-16** | **103** | 54 (53.8) | 15 (14.4) | 13 (12.5) | 8 (7.7) | 6 (5.8) | 2 (1.9) | 4 (3.9) | 0 | 1 (1) | 0 |
| **2016-17** | **114** | 45 (42.2) | 19 (16.4) | 17 (14.7) | 2 (1.7) | 12 (10.4) | 8 (6.9) | 7 (6) | 2 (1.7) | 2 (2) | 0 |
| **2017-18** | **105** | 39 (39.6) | 22 (20.7) | 11 (10.4) | 15 (14.2) | 4 (3.8) | 4 (3.8) | 3 (2.8) | 5 (4.7) | 1 (1) | 1 (33) |
| **2018-19** | **116** | 38 (34.7) | 16 (13.6) | 23 (19.5) | 16 (13.6) | 11 (9.3) | 4 (3.4) | 4 (3.4) | 2 (1.7) | 1 (1) | 1 (33) |
| **Co-infections, n (%)** | **239 (27)** | 63 (20) | 49 (32) | 38 (31) | 19 (22) | 25 (29) | 16 (41) | 12 (32) | 9 (53) | 5 (56) | 3 (100) |
| **Upper respiratory sample, n (%)** | **809 (92.5)** | 297 (92) | 140 (93) | 117 (95) | 83 (95) | 78 (92) | 35 (90) | 33 (87) | 16 (94) | 8 (89) | 2 (66) |
| **Lower respiratory sample, n (%)** | **32 (3.7)** | 11 (3) | 5 (3) | 4 (3) | 2 (5) | 1 (1) | 2 (5) | 5 (13) | 0 | 1 (11) | 1 (33) |
| **Upper + Lower respiratory sample, n (%)** | **34 (4)** | 15 (5) | 6 (4) | 2 (2) | 2 (5) | 6 (7) | 2 (5) | 0 | 1 (6) | 0 | 0 |
| **Shedding, mean day (SD)** | **26.6 (20.4)** | 27.2 (17.9) | 34,2 (21.6) | 23.3 (13.2) | 18.6 (24.9) | 32.5 (29.8) | 21.8 (14.8) | 7 | 10 (11.3) | NA | NA |
| **First RVI, post, mean day (SD)** | **334 (338)** | 227 (262) | 364 (369) | 382 (340) | 371 (323) | 409 (363) | 503 (418) | 279 (340) | 501 (435) | 394 (432) | 95 (31) |

RVI= respiratory viral infection; HSCT= hematopoietic stem cell transplant; hRV =human rhinovirus; hCoV=human coronavirus; PIV=parainfluenza virus; FLU=influenza; RSV=respiratory syncytial virus; hMPV=human metapneumovirus; ADV=adenovirus; Boca=bocavirus; EV=enterovirus; PEV=parechovirus; n=number; SD=standard deviation; Tx= transplantation; IQR=interquartile rang
